# Socioeconomic Factors Associated With a Late-Stage Pancreatic Cancer Diagnosis: An Analysis of the National Cancer Database

**DOI:** 10.7759/cureus.35857

**Published:** 2023-03-07

**Authors:** Jillian M Gallegos, Alexandra Taylor, Victoria Vardell, Peter T Silberstein

**Affiliations:** 1 Oncology, Creighton University School of Medicine, Omaha, USA; 2 Internal Medicine, University of Utah, Salt Lake City, USA

**Keywords:** health disparities, delayed diagnosis, socioeconomic, pancreatic cancer, ncdb

## Abstract

Background

Pancreatic adenocarcinoma is an aggressive, lethal cancer. It is the fourth leading cause of cancer death in the United States and is often asymptomatic until later stages. Thus, it is critical to identify patients earlier in their disease course. Socioeconomic factors can assist in determining who is at higher risk of presenting at later stages of the disease. Using the National Cancer Database (NCDB), we aim to identify the associations between socioeconomic factors and the stage of pancreatic cancer at diagnosis.

Methodology

In this study, 256,822 patients from the NCDB who were diagnosed with pancreatic cancer from 2004 to 2018 at stage 0-I and stage IV were compared based on age, race, sex, ethnicity, insurance type, income, geographic location, education, and Charlson-Deyo score. Demographic factors of patients who presented with early and late-stage disease were compared using the chi-squared test and multivariate logistic regression.

Results

We identified significant associations between race, sex, insurance status, education, income, and geographic location with the stage of disease at diagnosis. Males were more likely to be diagnosed with late-stage cancer than early-stage (52.8% vs. 47.9%, p < 0.001). Females were more likely to have an earlier-stage diagnosis when compared to males (odds ratio (OR) = 0.857, 95% confidence interval (CI) = 0.839-0.875, p < 0.001). Black patients presented at a later stage when compared to White patients (OR = 1.106, 95% CI = 1.069-1.144, p < 0.001). Private and Medicaid insurance had higher rates of late-stage diagnosis than early stages, and all other types of insurance had lower rates of late-stage diagnosis than patients without insurance (p < 0.001). Patients from a zip code with less than $38,000 median household income and zip codes with lower levels of high school graduation had higher rates of late-stage diagnosis (p < 0.025).

Conclusions

Factors associated with the increased likelihood of pancreatic cancer presentation at the advanced stage compared to the early stage include multiple minority and traditionally underserved populations. Black race, underinsurance, or residing in low-income or low-education zip codes was significantly associated with presenting at a late stage, which is strongly associated with worse survival outcomes.

## Introduction

Pancreatic cancer is an aggressive, rapidly progressing cancer with poor overall survival and is the fourth leading cause of cancer death in the United States [1]. Most commonly diagnosed in men between the ages of 40-85, with a higher risk in smokers and obese patients, the full etiopathogenesis of pancreatic cancer remains insufficiently understood. These tumors are often asymptomatic in early stages, and without any screening recommendations currently in place, 80-85% of these malignancies are diagnosed at a late stage [2,3]. Early surgical resection is the most promising and only potentially curative treatment for long-term survival in pancreatic cancer [4]. There are no curative options in patients with unresectable disease, and methods employed in these patients, including radiation and chemotherapy, have limited efficacy in improving long-term survival [5]. Due to its highly malignant nature, overall survival remains poor, with current five-year survival estimates of around 6% in patients diagnosed with unresectable and late-stage disease [6].

With a successful cure and long-term survival of pancreatic cancer relying on early diagnosis, an understanding of the socioeconomic factors associated with the later stage of diagnosis is necessary to assist in programs to improve early diagnosis, and, as a result, long-term survival. While socioeconomic disparities among pancreatic cancer patients undergoing curative resection have been analyzed, the socioeconomic factors of patients presenting with stage IV cancer versus stage I at diagnosis have not been analyzed [4]. The goal of our study is to assess any significant socioeconomic and demographic factors that are associated with a delay in diagnosis, resulting in increased rates of diagnosis of unresectable and advanced pancreatic cancer.

This article was previously presented as a meeting abstract at the 2022 American Society of Clinical Oncology Annual Meeting on June 4, 2022.

## Materials and methods

Patient selection

Patients diagnosed with pancreatic cancer were identified from the National Cancer Database (NCDB) using the International Classification of Diseases for Oncology, Third Edition (ICD-O-3) morphology codes 8150/3 and 8154/3. Patients were included if they were diagnosed at stage 0-1 or stage 4, classified by NCDB analytic stage group from the Seventh Edition AJCC staging for pancreatic cancer. Patients who fell in the middle stages (II-III) were excluded to examine relationships between the extremes in stages of diagnosis and socioeconomic status (SES) indicators. The NCDB is sponsored by the American Cancer Society and the American College of Surgeons to provide data on cancer patients treated at Commission on Cancer (CoC)-accredited facilities [7]. Data from over 70% of new cancer diagnoses in the United States are collected and reported in the NCDB [7]. All patient information is deidentified but reports on a variety of factors including the stage of diagnosis, socioeconomic factors, treatment methods, and overall survival. Data of patients with pancreatic adenocarcinoma from 2004 to 2018 was requested from the NCDB via a participant user file.

Patient characteristics

Patients were excluded if they did not meet AJCC staging criteria or if the stage was unknown. In patients who met the inclusion criteria, patients diagnosed at stage 0-1 and stage IV were compared by sex, age at diagnosis, race, Hispanic/Spanish origin, insurance status, geographic location in the United States, Charlson-Deyo comorbidity score, education, and income of residential zip code. Race in the NCDB is patient-identified and was categorized into four groups: White, Black, American Indian, and Other. Hispanic/Spanish origin was divided into two groups: Non-Hispanic and Hispanic. Geographic location was divided into four broad regions of the United States: Northeast/Atlantic, South, West, and Midwest. Income was categorized by the median household income from 2008 to 2012 for the zip code in which the patient resided at the time of diagnosis and provided by quartile compared to 2012 US Census data. Similarly, education level was measured via the percentage of residents in the patient’s zip code who did not graduate from high school from 2008 to 2012 by 2012 US census data. The Charlson-Deyo comorbidity score is an additive score based on pre-existing comorbidities and provided as scores of 0, 1, 2, or ≥3. Insurance status was divided into five groups consisting of no insurance, private, Medicaid, Medicare, and other government insurance.

Statistical analysis

SPSS Statistics version 27 (IBM Corp., Armonk, NY, USA) was used to analyze the association between the stage of pancreatic adenocarcinoma at diagnosis with age, sex, race, ethnicity, Charlson-Deyo score, income, education, geographic location, and insurance status. Early-stage diagnoses were defined as NCDB Analytic Stage group 0-I, and late-stage diagnoses were NCDB Analytic Stage IV. The chi-squared test was used to assess the significance (p < 0.05) of demographic factors and stage of diagnosis. A multivariable binary logistic regression model was used to identify socioeconomic variables that indicated later stages of disease at diagnosis. Sex, age, race, Hispanic origin, location, insurance, income, education, and Charlson-Deyo comorbidity were all variables included in the multivariate analysis.

Descriptive statistics were conducted using SPSS for Windows, version 27.0 (IBM Corp., Armonk, NY, USA). The multivariate analysis was conducted using SAS version 9.4 (SAS Institute Inc., Cary, NC, USA).

## Results

The descriptive statistics for the cohort of 256,822 patients with pancreatic adenocarcinoma are displayed in Table 1. The majority of patients (78.2%) presented with stage IV disease. The cohort had a slight male predominance, with most patients being White (83.5%) and of non-Hispanic ethnicity (94.3%). Most patients had Charlson-Deyo comorbidity scores of 0 (65.1%), Medicare insurance (58.6%), and were from the Northeast/Atlantic US region (44.5%).

**Table 1:** Descriptive statistics for the cohort of 256,822 patients with pancreatic adenocarcinoma from the National Cancer Database (2004-2018).

Variable	Stage 0-I: % of stage group	Stage IV: % of stage group	Total	P-value
Sample size	55,780	201,042	256,822	
Age (years)				
0–24	245 (0.4%)	119 (0.1%)	364 (0.1%)	<0.001
25–49	4,214 (7.6%)	12,270 (6.1%)	16,484 (6.4%)	<0.001
50–74	29,957 (53.7%)	124,613 (62.0%)	154,570 (60.2%)	<0.001
≥75	21364 (38.3%)	64040 (31.9%)	85404 (33.3%)	<0.001
Sex				
Male	26,703 (47.9%)	106,211 (52.8%)	132,914 (51.8%)	<0.001
Female	29,077 (52.1%)	94,831 (47.2%)	123,908 (48.2%)	<0.001
Race				
Caucasian	46,443 (84.2%)	165,805 (83.3%)	212,248 (83.5%)	<0.001
African American	6,348 (11.5%)	25,716 (12.9%)	32,064 (12.6%)	<0.001
American Indian	168 (0.3%)	536 (0.3%)	704 (0.3%)	<0.001
Other	2,230 (4.0%)	7,020 (3.5%)	9,250 (3.6%)	<0.001
Spanish/Hispanic origin				
Non-Hispanic	50,801 (94.5%)	180,349 (94.2%)	231,150 (94.3%)	0.06
Hispanic	2,930 (5.5%)	11,027 (5.8%)	13,957 (5.7%)	0.06
Charlson-Deyo score				
0	36,214 (64.9%)	130,954 (65.1%)	167,168 (65.1%)	0.007
1	13,163 (23.6%)	47,766 (23.8%)	60,929 (23.7%)	0.007
2	3,934 (7.1%)	13,345 (6.6%)	17,279 (6.7%)	0.007
≥3	2,469 (4.4%)	8,977 (4.5%)	11,446 (4.5%)	0.007
Insurance status				
None	1,088 (2.0%)	6,417 (3.3%)	7,505 (3.0%)	<0.01
Private	16,524 (30.2%)	62,620 (31.8%)	79,144 (31.4%)	<0.01
Medicaid	2,561 (4.7%)	12,020 (6.1%)	14,581 (5.8%)	<0.01
Medicare	33,735 (61.6%)	113,857 (57.8%)	147,592 (58.6%)	<0.01
Other government	849 (1.6%)	2,163 (1.1%)	3,012 (1.2%)	<0.01
Level of education (% of no high school diploma 2008–2012)				
≥21.0%	8,431 (16.6%)	32,630 (17.2%)	41,061 (17.0%)	0.003
13.0–20.9%	12,853 (25.3%)	48,611 (25.6%)	61,464 (25.5%)	0.003
7.0–12.9%	16,866 (33.2%)	62,332 (32.8%)	79,198 (32.9%)	0.003
<7.0%	12,676 (24.9%)	46,572 (24.5%)	59,248 (24.6%)	0.003
Median household income (quartiles 2008–2012)				
8,660 (17.0%)	33,484 (17.6%)	42,144 (17.5%)	0.025
$38,000–$47,999	11,679 (23.0%)	43,212 (22.7%)	54,891 (22.8%)	0.025
$48,000–$62,999	13,565 (26.7%)	50,488 (26.6%)	64,053 (26.6%)	0.025
≥$63,000	16,899 (33.3%)	62,849 (33.1%)	79,748 (33.1%)	0.025
Geographic location				
Northeast	23,439 (43.2%)	89,142 (44.8%)	112,581 (44.5%)	<0.001
South	8,674 (16.0%)	27,700 (13.9%)	36,374 (14.4%)	<0.001
Midwest	13,292 (24.5%)	50,351 (25.3%)	63,643 (25.1%)	<0.001
West	8,851 (16.3%)	31,625 (15.9%)	40,476 (16.0%)	<0.001

Male, Black, and uninsured, or insurance with private or Medicaid insurance, were all associated with increased odds of late-stage pancreatic cancer diagnoses (Table 1). Patients from areas with lower levels of education (≥13% zip code with no high school diploma) and lower median household incomes (<$38,000) were also associated with increased rates of stage IV diagnoses. The chi-squared analysis showed no significant association in Hispanic/Spanish origin with regard to the stage of pancreatic cancer at diagnosis. Patients from the Midwest and Northeast also reported higher rates of stage IV cancer at diagnosis. The Charlson-Deyo score of 2 was the only comorbidity rating to have a higher presentation of early-stage disease. Female patients, White race, Charlson-Deyo comorbidity scores of 2, Medicare insurance, higher levels of education, and median household income were all associated with the earlier stage of disease at diagnosis. Geographic regions including the West and South also reported an increased percentage of patients presenting with early-stage disease.

On multivariate analysis, as seen in Table 2, adjusted for sex, comorbidity score, race, ethnicity, insurance, and location as well as income and education level of zip code, patients aged 50-74 were associated with a 30% increased odds of a late-stage pancreatic cancer diagnosis (odds ratio (OR) = 1.306, 95% confidence interval (CI) = 1.246-1.372, p < 0.001). Compared to males, females were 15% less likely to receive a delayed diagnosis (OR = 0.857, 95% CI = 0.839-0.875, p < 0.001). Black patients had 10% increased odds of an advanced diagnosis (OR = 1.106, 95% CI = 1.069-1.144, p < 0.001), and Other races had 15% increased odds of an early diagnosis when compared to White patients (OR = 0.849, 95% CI = 0.804-0.897, p < 0.001). The multivariable logistic regression did find that Hispanic patients had a significant 5% increase in odds of stage IV diagnosis compared to their non-Hispanic counterparts (OR = 1.052, 95% CI = 1.003-1.104, p<0.039). Charlson-Deyo comorbidity scores of 2 had lower odds of delayed diagnosis by 5% (OR = 0.948, 95% CI = 0.910-0.988, p < 0.011), and all types of insurance had significantly decreased odds of a stage IV diagnosis compared to patients without insurance (up to 60% for those with other government insurance) (p < 0.001). Higher levels of education in the patient’s zip code were associated with early-stage diagnosis (p < 0.002). There was no significance in the median household income of the patient on multivariate analysis. Finally, patients in the South and West had lower odds of advanced diagnosis compared to patients in the Northeast (OR = 0.786, 95% CI = 0.762-0.811, p < 0.001 and OR = 0.945, 95% CI = 0.917-0.974, p < 0.001, respectively).

**Table 2:** Multivariate analysis of the cohort of 256,822 patients with pancreatic adenocarcinoma from the National Cancer Database (2004-2018).

Variable	Odds ratio (95% confidence interval)	P-value
Age (years)		
0–49	Reference group	
50–74	1.308	<0.001
≥75	0.991	0.727
Sex		
Male	Reference group	
Female	0.857	<0.001
Race		
Caucasian	Reference group	
African American	1.106	<0.001
American Indian	0.920	0.403
Other	0.849	<0.001
Spanish/Hispanic origin		
Non-Hispanic	Reference group	
Hispanic	1.052	0.039
Charlson-Deyo Score		
0	Reference group	
1	0.991	0.490
2	0.948	0.011
≥3	0.974	0.299
Insurance status		
None	Reference group	
Private	0.629	<0.001
Medicaid	0.802	<0.001
Medicare	0.591	<0.001
Other government	0.417	<0.001
Level of education (% of no high school diploma 2008–2012)		
≥21.0%	Reference group	
13.0–20.9%	0.971	0.103
7.0–12.9%	0.941	0.002
<7.0%	0.930	0.001
Median household income (quartiles 2008–2012)		
Reference group	
$38,000–$47,999	0.987	0.476
$48,000–$62,999	1.006	0.742
≥$63,000	1.038	0.081
Geographic location		
Northeast	Reference group	
South	0.786	<0.001
Midwest	1.002	0.904
West	0.945	<0.001

## Discussion

Our study demonstrates the increased odds of presenting with stage IV pancreatic cancer in a number of traditionally underserved patient populations, thus illustrating the ever-present disparities in healthcare in the United States. Black patients were shown to have a 10% increased likelihood of advanced cancer diagnosis compared to White patients. This is notable as previous studies have demonstrated up to a 90% higher incidence of pancreatic cancer in Blacks compared to other races, and poorer overall prognosis for these patients [8]. Hispanic patients were also found to have increased odds of late-stage diagnosis compared to non-Hispanics. Indicators of lower SES (lower education levels, lower median household income, and lack of insurance) were also shown to have increased odds of delayed pancreatic cancer diagnoses. These findings are consistent with analyses in other cancer databases that found increased rates of distant-stage cancer diagnosis among more impoverished counties in the United States, specifically among cancers without routine screening methods [9]. These findings reaffirm the notion that economic status does play an important role in healthcare and outcomes, and that healthcare disparities are a continuing challenge being faced in US medicine [10].

Our research illustrates how underserved groups affected by lower SES, lack of higher-level education, and racial and ethnic minority discrimination suffer the cost of healthcare disparities with more advanced prognoses at diagnosis. Social, racial, and economic disparities have significant and real effects on healthcare outcomes, especially for cancers such as pancreatic cancer with devastating late-stage prognoses. This calls attention to how we, as healthcare providers, can reduce these disparities and improve early diagnosis in groups from lower SES.

Currently, there are no recommended screening methods for pancreatic cancer, and literature has suggested that screening of asymptomatic, average-risk individuals may cause more harm than benefit [11]. Without reliable screening methods, healthcare providers are called to decrease healthcare disparities through the reduction of structural racism in medicine and by increasing healthcare literacy for susceptible populations. Smoking cigarettes is one of the few well-known risk factors for pancreatic cancer that has been shown to double the risk, thus physicians should make a concerted effort to educate and encourage smoking cessation in their patients, specifically those at higher risk [12].

Further, our study also shows that older males are more likely to present with later stages of pancreatic cancer relative to females. In general, males are more likely to get pancreatic cancer as the “prototypical patient” with pancreatic cancer is an older, White male. Despite the lack of screening methods, doctors should be cognizant to consider testing for pancreatic cancer when suspicious in older populations of all sexes and races and to encourage smoking cessation among all patients as well. In fact, rates of pancreatic cancer in males overall are declining, likely due to generational changes in smoking habits in recent years [12]. Regardless, it is imperative that physicians encourage all patients to stop smoking as the overall benefits of smoking cessation span far beyond that of lowering the risk of pancreatic cancer.

It is important that further research investigate whether reducing modifiable risk factors, such as smoking and obesity, does in fact reduce the incidence of pancreatic cancer or improve survivability in these populations with other non-modifiable risk factors such as age, race, and sex. Moreover, further retrospective and cross-sectional research should be done to elucidate other not well-known modifiable risk factors that may be contributing to the development of pancreatic cancer. Better guidelines for the qualification of high-risk patients should be developed, with the inclusion of modifiable and non-modifiable (demographic) risk factors, so that non-invasive screening methods (via ultrasound or CT scan) may one day be employed in appropriate patients who are at the highest risk as stratified by both socioeconomic and other risk factors. By improving guidelines to screen high-risk patients for pancreatic cancer, more cases may be found in the early, curative phases via surgical resection, thus improving overall survival and prognosis for these patients.

Our data show overall much higher reporting from the Northeastern/Atlantic United States. This may be in part due to the fact that these states are highly populous and include the entire Atlantic seaboard. Moreover, many big academic medical institutions are located in the Northeast, which may lead to higher rates of pancreatic diagnoses at these institutions and a greater number of patients who may travel to these institutions for treatment due to their prestige and research in the areas of oncology. Literature has shown that throughout the United States, rates of pancreatic cancer are higher in urban areas [13], and there are a greater number of pancreatic cancer clinical trials in the Northeast United States [14]. It is also possible that patients in rural areas who have lower access to healthcare with fewer medical centers and physicians readily available may not seek medical care that would provide them with a pancreatic cancer diagnosis due to the far travel and costs required to reach such medical access. These factors may also explain higher rates of reporting in the Northeast/Atlantic United States.

Our study had a relatively large sample size due to the abundance of pancreatic cancer in the United States; however, it did have limitations. NCDB accounts for only approximately 70% of new cancer diagnoses in the United States so any cancers diagnosed at non-CoC-accredited facilities do not get reported to the NCDB database [15]. Thus, while NCDB is an incredibly useful tool, it is not truly a population-based analysis as patients must receive care from a hospital that participates in the registry [15]. Further, a large number of patients in the NCDB database were not assigned an AJCC stage group or were not applicable for staging, thus decreasing the number of patients who were analyzed. However, our data have been consistent with results from the literature using other cancer databases which provides support to our conclusions.

## Conclusions

Multiple socioeconomic and demographic factors were associated with an increased likelihood of a late-stage pancreatic cancer diagnosis. Sex, age, race, Hispanic/Spanish origin, Charlson-Deyo comorbidity score, insurance status, education level, and geographic location all had significant findings in the increased likelihood of a delayed diagnosis. Notably, African Americans, Hispanic patients, and patients without insurance all had higher odds of an advanced diagnosis. As some groups are at increased risk of presenting with later-stage disease, it may be important to assess current health disparities in certain groups and assess how these gaps in healthcare can be minimized via changes in clinical practice.
